# Stereopsis outcome in refractive accommodative esotropia successfully aligned by full hypermetropic correction

**DOI:** 10.1007/s10384-025-01181-8

**Published:** 2025-04-18

**Authors:** Teiji Yagasaki, Yoshimi Yokoyama, Ayaka Yagasaki, Kenta Hozumi

**Affiliations:** 1 Yagasaki Eye Clinic, 62-6 Gonaka, Kaimei, Ichinomiya, Aichi 494-0001 Japan; 2 Department of Ophthalmology, Japan Community Health Care Organization Chukyo Hospital, Nagoya, Aichi 457-8510 Japan

**Keywords:** Stereopsis, Refractive accommodative esotropia, Onset of misalignment, Duration of misalignment

## Abstract

**Purpose:**

To investigate factors associated with stereopsis outcomes in refractive accommodative esotropia (RAET) successfully aligned by full hypermetropic correction.

**Study design:**

Retrospective.

**Methods:**

In 171 patients with RAET, ages at onset, initial visit, and first use of glasses, duration of misalignment, cycloplegic refraction, and ocular deviation were compared between groups based on stereopsis outcome: Group F, fine stereopsis ≤60"; Group C, coarse stereopsis >60" but ≤3000"; and Group N, nil stereopsis.

**Results:**

Groups F, C and N comprised 37, 82 and 52 patients, respectively. Although no differences in initial cycloplegic refraction or duration of misalignment were seen between groups, earlier ages at onset, initial visit, and first use of glasses were associated with stereopsis outcome. Final near deviation with glasses also affected fine stereopsis outcome. Groups F, C and N among the 68 patients with onset at >2 years comprised 29, 39, and 0 patients, respectively. In contrast, the 58 patients with onset at ≤1 year and the 45 patients with onset at >1 but ≤2 years showed significantly worse incidences (Groups F, C and N: 5, 18 and 35 patients and 3, 25 and 17 patients, respectively; p<0.001 each, chi-squared test). In the 103 patients with onset ≤2 years, duration of misalignment ≤4 months was associated with significantly better stereopsis outcome than duration of misalignment >4 months (68% vs 42%, p=0.032; Fisher’s exact test).

**Conclusions:**

The most important factor associated with stereopsis outcome was earlier age at onset, with the worst outcome for children with age at onset ≤1 year.

## Introduction

Accommodative esotropia is one of the most common forms of esotropia encountered in childhood, usually arising between 6 months and 7 years of age, with a mean age at onset of 2.5 years [1–3]. This convergent deviation is caused by the efforts of the eyes to focus clearly with activation of the accommodative reflex [4]. Accommodative esotropia is classically divided into three types: refractive accommodative esotropia (RAET); non-refractive accommodative esotropia; and partially accommodative esotropia. As the accommodative convergence/accommodation (AC/A) ratio in RAET is ≤5, the angle of esodeviation is equal for distance and near, and resolves within orthophoria or 10 prism diopters (PD) with glasses to correct full hyperopia or hyperopic astigmatism [5].

Most patients with RAET achieve favorable outcomes in binocularity, since esodeviation usually develops after 2 years old, by which time significant maturation of stereopsis has occurred [6]. However, not all patients with RAET display normal stereopsis even after successful realignment with glasses [7–17]. Several factors, including earlier onset [7, 9, 10], longer duration of esodeviation [9, 11], anisometropia [12, 15] or amblyopia [9, 12], residual esodeviation [11, 14] and poor compliance with wearing glasses [16, 17], are reportedly associated with poor stereopsis.

Among patients with RAET, cases with onset prior to 6 months to 1 year old were reported [3, 18, 19]. Fawcett and coworkers demonstrate that patients who showed intermittent misalignment or constant misalignment lasting <4 months achieved better stereopsis than those with constant misalignment for ≥4 months, even if age at onset in those patients was ≤2 years [11]. However, mean duration between age at onset or diagnosis and initial correction with glasses was from 4 months to 4–5 years. Such onset is before significant maturation of stereopsis and this duration seems critical to the development of stereopsis, particularly among patients with onset before 6–12 months old [6, 10].

Recent studies have investigated associations between duration of misalignment and binocular sensory outcomes in patients with esotropia, and very early surgical alignment before 6–8 months old can play an important role in achieving better stereopsis for cases of infantile esotropia [6, 20]. RAET can arise within a relatively wide period, between 6 months and 7 years old, and age at onset in particular may influence stereopsis outcomes. The purpose of this study was to clarify factors associated with better stereopsis outcomes among patients with RAET and the influences of age at onset, age at initial visit, age at alignment, duration of misalignment, and duration of treatment on random dot stereoacuity outcomes in patients with RAET.

## Subjects and methods

The medical records of all 345 patients diagnosed with accommodative esotropia with a minimum observation period of 6 months after initiation of prescribed correction attending the Japan Community Health Care Organization Chukyo Hospital or Yagasaki Eye Clinic between January and December 2023 were retrospectively reviewed.

At the initial visit, atropine sulfate 1% was prescribed to all patients with esotropia. Within 1 week after the initial visit, cycloplegic refraction using atropine sulfate 1% once a day for 3 days was performed as part of a routine, comprehensive evaluation. After full hyperopic or hyperopic astigmatic correction, RAET was diagnosed when residual esodeviation for distance and near with glasses was within orthophoria or 10 PD. As routine examinations, manifest refraction was repeated at every visit and cycloplegic refraction was repeated at intervals of 6–12 months to adjust correction of glasses as necessary. After examining the medical records of all 345 patients, 107 patients diagnosed with partially refractive accommodative esotropia and 36 patients diagnosed with non-refractive accommodative esotropia with high AC/A ratio were retrospectively excluded during the observation period.

Patients with final visual acuity <1.0 were excluded to eliminate the influence of partially cured amblyopia on stereopsis. Patients were also excluded if they had any history of previous strabismic surgery, developmental delay, preterm birth (at ≤37 weeks of gestation), Down syndrome, or neurologic or ocular abnormalities other than esotropia. During follow-up, patients showing deterioration in ocular deviation as consecutive exotropia were also excluded to eliminate influences on stereopsis outcome. Finally, a total of 171 patients diagnosed with RAET were included in this study.

The angle of deviation was primarily assessed using only the alternate prism cover test. Krimsky and Hirschberg methods were not applied, to avoid inaccuracies in the angle [21]. For patients too young for distance testing, measurements were carried out for near only. Each patient underwent at least three measurements by one of the authors on at least three separate examinations within a few weeks until reliable measurements were achieved.

Best-corrected visual acuity was measured using the Landolt C in older children and by Teller Acuity Cards or Cardiff Acuity Cards in infants and younger children. Amblyopia was defined as a difference of ≥2 Landolt C lines, or a difference of ≥2 octaves in grating acuity between the best-corrected visual acuity of two eyes.

Binocular response was evaluated by the Bagolini striated lenses test at distance and at near. Responses were classified as positive if two crossed streaks with or without a central scotoma were seen, and as negative if both were never seen at the same time [22, 23]. Stereopsis was measured using the Titmus Stereo Tests (Stereo Optical) or Randot stereotest (Stereo Optical). In cases showing differing stereopsis between those two tests, the better stereopsis value was used for analysis. Stereopsis ≤60 arcsec (") on the Titmus circles’ test or Randot graded circle test was defined as fine stereopsis, and coarse stereopsis was defined as stereopsis >60" to ≤3000" [6, 8, 24]. In cases with unmeasurable stereopsis, stereopsis was recorded as nil, and results were evaluated as 10,000" for proper statistical assessment [9]. All values of stereopsis were additionally transformed to the logarithm of arc seconds for the purpose of analysis.

Age at onset was determined by both the clinical history of first intermittent misalignment and evidence of these changes in previous photographs of patients. The following parameters were retrospectively reviewed for statistical analyses: age at onset (intermittent phase); age at initial visit; age at first wearing glasses; age at final visit; duration of misalignment (from first reported onset to first wearing glasses); duration of treatment; sex, cycloplegic refraction (spherical-equivalents); deviation at distance and near fixations without glasses at initial visit and with glasses at final visit; poor compliance with wearing glasses; and stereopsis and binocular response after refractive corrections.

Compliance with wearing glasses was categorized based on the definition by Hussein et al. [6], as follows:

- Good: patient arrived at the clinic wearing glasses for ≥75% of visits.
- Fair: patient arrived at the clinic wearing glasses for >25% but <75% of visits.
- Poor: patient arrived at the clinic wearing glasses for ≤25% of visits.

This study was approved by the institutional review board of the Japan Community Health Care Organization Chukyo Hospital. Informed consent was obtained from the parents or guardians of each patient. All study and data collection protocols conformed to all local laws and complied with the principles of the Declaration of Helsinki.

Collected data were entered into an Excel spreadsheet (Microsoft,). Mean values for subgroups of stereopsis outcomes were compared using a two-tailed *t* test, the chi-squared test, Fisher’s exact test or one-way analysis of variance (ANOVA). In positive one-way ANOVA results, multiple comparisons were performed using the Kruskal–Wallis test or the Bonferroni test. For statistical analyses, p-values of <0.05 were considered statistically significant. With the Bonferroni test, two-tailed p-values of <0.05/3 were also considered statistically significant.

## Results

A total of 171 patients (81 boys, 90 girls) with RAET were included in this study. Although some patients were unable to wear glasses during part of the waking hours at home as reported by parents, compliance with wearing glasses during 80 % or more of the waking hours was evaluated as good in all patients.

Table 1 shows the background characteristics of patients. Mean value of final stereopsis for all patients was 613" (2.8 ± 1.0 log arcsec) and patients were divided into three groups based on the degree of final stereopsis: Group F, fine stereopsis (37 patients, 22%); Group C, coarse stereopsis (82 patients, 48%); and Group N, absence of stereopsis (52 patients, 30%).

**Table 1:** Characteristics of all patients with refractive accommodative esotropia

n=171 (male/female: 81/90)	Mean		SD
Age at onset (years)	2.0	±	1.5
Age at initial visit (years)	3.1	±	1.9
Initial cycloplegic refraction (D)			
Right	+4.5	±	2.0
Left	+4.6	±	2.0
Initial uncorrected deviation (PD)			
At near	20.4	±	8.9
At distance (N:151)	20.0	±	7.4
Age at first wearing glasses (years)	3.1	±	1.9
Duration of misalignment (years)	1.1	±	1.1
Duration of treatment (years)	6.5	±	3.6
Age at final visit (years)	9.7	±	4.1
Final cycloplegic refraction (D)			
Right	+4.2	±	2.1
Left	+4.4	±	2.1
Final deviation with glasses (PD)			
At near	2.2	±	3.4
At distance	2.2	±	4.1
Final stereopsis (log arcsec)	2.8	±	1.0
(Mean stereopsis (arcsec))	(613”)		

*SD* standard deviation, *D* diopter, *PD* prism diopters

**Fig. 1:**
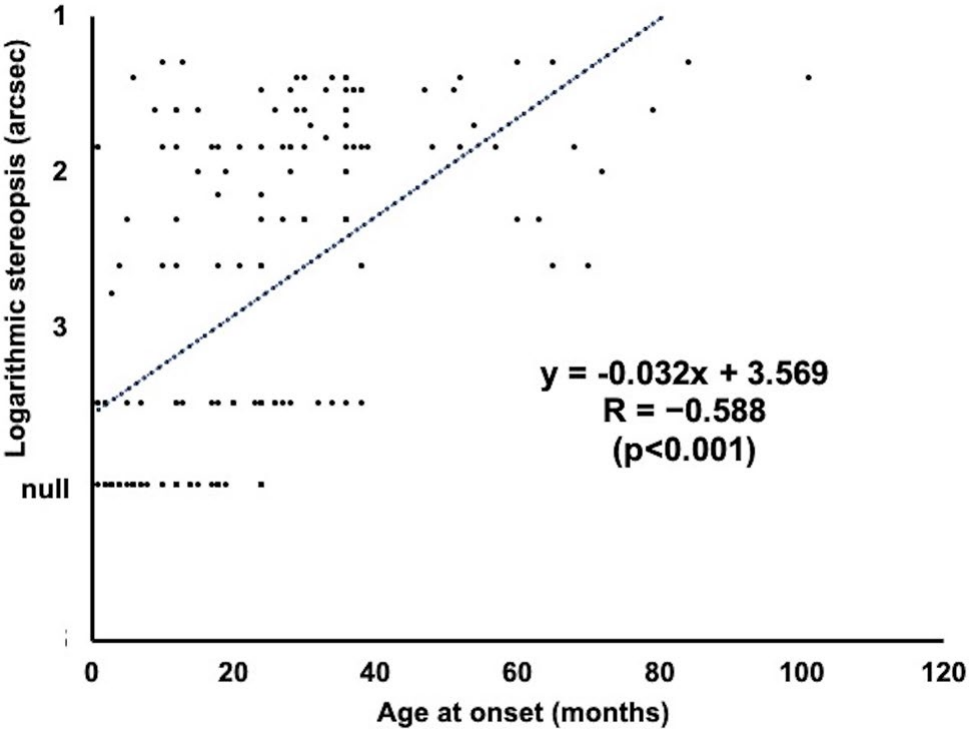
Relationship between age at onset (months) and stereopsis outcome (arcsec). For statistical assessment, the logarithmic value of stereopsis is evaluated as 10,000 arcsec in patients with absence of stereopsis. Trendline analysis reveals a significant correlation (y = −0.032x + 3.569; R=−0.588; p<0.001)

**Fig. 2:**
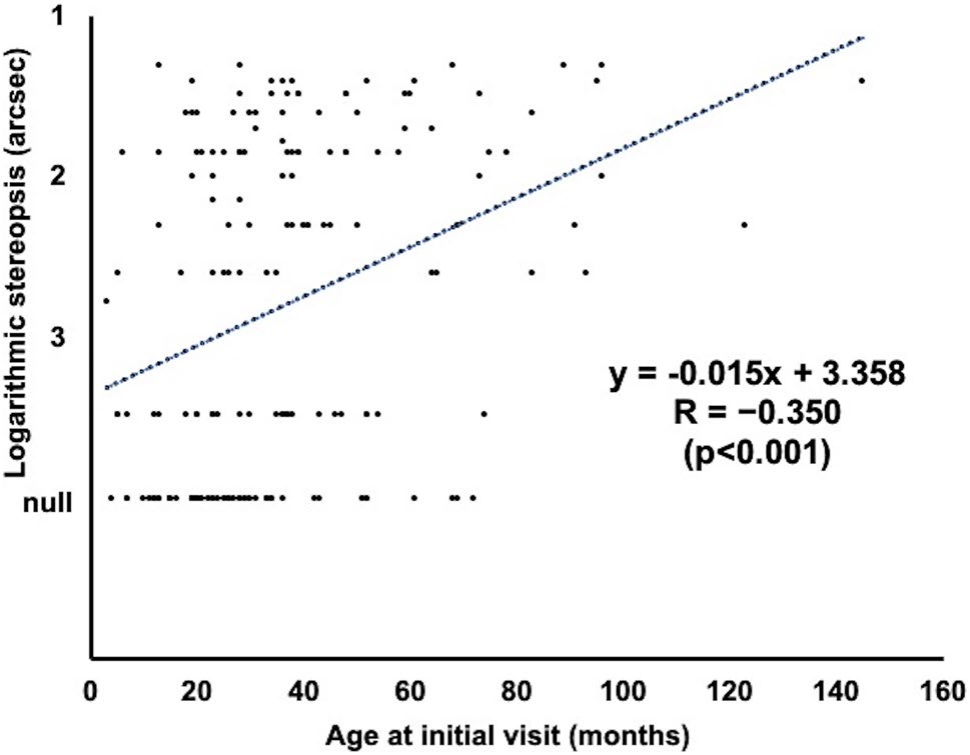
Relationship between age at initial visit (months) and stereopsis outcome (arcsec). For statistical assessment, logarithmic value of stereopsis is evaluated as 10,000 arcsec in patients with absence of stereopsis. Trendline analysis reveals a significant correlation (y = −0.015x + 3.358; R=−0.350; p<0.001)

**Fig. 3:**
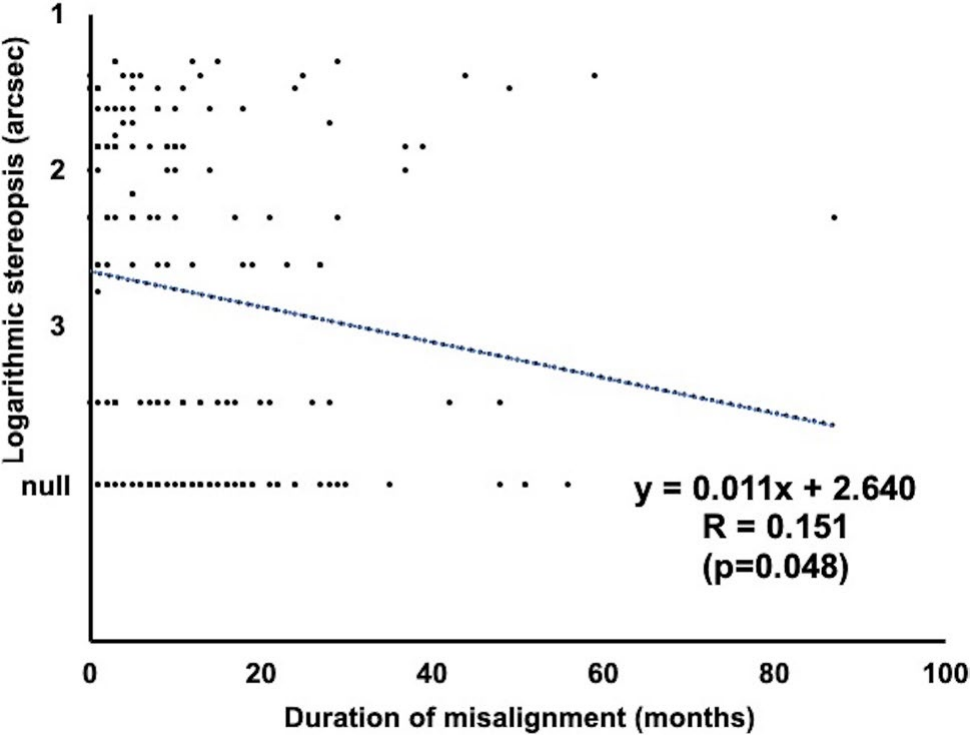
Relationship between duration of misalignment (months) and stereopsis outcome (arcsec). For statistical assessment, logarithmic value of stereopsis is evaluated as 10,000 arcsec in patients with absence of stereopsis. Trendline analysis reveals a significant correlation (y = 0.011x + 2.640; R=0.151; p=0.048)

Table 2 compares clinical variables between groups. Although the difference in the angle of initial uncorrected deviation at near was significant only between Groups F and N, no significant differences were seen in angle of initial uncorrected deviation at distance or in initial cycloplegic refraction of both eyes between any of the three groups. In other words, refractive errors and angle of deviation with glasses showed no association with stereopsis outcomes. On the other hand, earlier ages at onset, initial visit, and delayed initiation of wearing glasses were critical factors associated with worse outcomes of stereopsis and those findings were supported by significant relationships between age at onset, initial visit, or duration of misalignment and stereopsis outcome (Figs. 1, 2, 3).

**Table 2:** Comparisons of clinical variables between groups based on stereopsis outcome

Clinical variable Mean ± SD	F: fine stereopsis			C: coarse stereopsis			N: nil stereopsis			ANOVA
	n=37 (22%)			n=82 (48%)			n=52 (30%)			F vs C, F vs N, C vs N
Age at onset (years)	3.1	±	1.7	2.2	±	1.4	0.9	±	0.6	*p*=0.010, *p*<0.001, *p*<0.001
Age at initial visit (years)	4.0	±	2.3	3.2	±	1.9	2.3	±	1.3	*p*=0.054, *p*<0.001, *p*=0.002
Initial cycloplegic refraction (D)										
Right	+4.6	±	2.2	+4.6	±	2.0	+4.4	±	1.8	*p*=0.855, *p*=0.654, *p*=0.410
Left	+4.7	±	2.2	+4.7	±	2.1	+4.5	±	1.7	*p*=0.979, *p*=0.646, *p*=0.524
Initial anisometropia (D)	0.5	±	0.4	0.7	±	0.8	0.4	±	0.4	*p*=0.1252, *p*=0.395, *p*=0.020
Initial uncorrected deviation (PD)										
At near	17.7	±	8.0	20.2	±	8.8	22.8	±	9.3	*p*=0.140, *p*=0.007, *p*=0.109
At distance (n=36,71,44)	18.6	±	8.2	19.7	±	6.8	21.8	±	7.6	*p*=0.473, *p*=0.069, *p*=0.129
Age at first wearing glasses (years)	4.0	±	2.3	3.2	±	1.9	2.3	±	1.3	*p*=0.054, *p*<0.001, *p*=0.002
Duration of misalignment (years)	1.0	±	1.1	1.0	±	1.1	1.4	±	1.1	*p*=0.958, *p*=0.116, *p*=0.042
Duration of treatment (years)	7.7	±	3.6	6.3	±	3.8	5.7	±	2.9	*p*=0.059, *p*=0.007, *p*=0.2941
Age at final visit (years)	12.0	±	4.1	9.7	±	4.3	8.2	±	3.1	*p*=0.016, *p*<0.001, *p*=0.016
Final cycloplegic refraction (D)										
Right	+4.0	±	2.4	+4.2	±	2.1	+4.4	±	1.8	*p*=0.686, *p*=0.362, *p*=0.520
Left	+4.1	±	2.4	+4.4	±	2.1	+4.6	±	1.8	*p*=0.623, *p*=0.342, *p*=0.520
Final deviation with glasses (PD)										
At near	0.8	±	1.4	2.0	±	3.2	3.5	±	4.2	*p*=0.005, *p*<0.001, *p*=0.032
At distance	1.1	±	2.2	2.3	±	4.2	3.0	±	4.8	*p*=0.048, *p*=0.015, *p*=0.384

*SD* standard deviation, *D* diopter, *PD* prism diopters

Table 3 demonstrates the relationship between age at onset and outcomes of stereopsis. Mean stereopsis at final visit in the 58 patients with onset at ≤1 year old, the 45 patients with onset at >1 to ≤2 years old and the 68 patients with age at onset >2 years were 2581" (3.4 ± 0.9 log arcsec), 1403" (3.1 ± 0.9 log arcsec) and 104" (2.0 ± 0.6 log arcsec), respectively. A positive relationship between age at onset and stereopsis outcomes was calculated with one-way ANOVA. Although no difference in stereoacuity was evident between the 58 patients with onset at ≤1 year old and the 45 patients with onset at >1 to ≤2 years old (p=0.298), Bonferroni testing showed no differences in stereoacuity between the 58 patients with onset at ≤1 year old and the 68 patients with onset at >2 years, or between the 45 patients with onset at >1 to ≤2 years old and the 68 patients with onset at >2 years (p<0.001 each). Frequencies of Groups F, C and N among the 58 patients showing onset at ≤1 year old were 9% (5 patients), 31% (18 patients), and 60% (35 patients), respectively. Frequencies of Groups F, C and N among the 45 patients with onset at >1 to ≤2 years old were 6% (3 patients), 56% (25 patients), and 38% (17 patients), respectively. No significant differences were apparent between those two groups (p=0.140, Kruskal–Wallis test). However, among the 68 patients with onset at >2 years old, frequencies of Groups F, C and N were 43% (29 patients), 57% (39 patients), and 0% (0 patients), respectively. Those numbers were significantly better than seen in patients showing onset at ≤1 year old or >1 to ≤2 years old (p<0.001 each). From these results, the most important factor associated with better outcome of stereopsis appeared to be onset at >2 years old. In general, if onset is delayed, initial diagnosis and treatment are likewise delayed.

**Table 3:** Comparisons of stereopsis outcomes between groups based on age at onset

Age at onset	Stereopsis	F: fine stereopsis	C: coarse stereopsis	N: nil stereopsis
	(log arcsec: mean ± SD)	(n=37)	(n=82)	(n=52)
≤1 y (n=58)	2581” * (3.4 ± 0.9)	5 (9%)	18 (31%)	35 (60%)
>1 to ≤ 2 y (n=45)	1403” ** (3.1 ± 0.9)	3 (6%)	25 (56%)	17 (38%)
> 2 y (n=68)	104” *** ( 2.0 ± 0.6)	29 (43%)	39 (57%)	0 (0%)

*SD* standard deviation, *y* yearsr

*p*=0.298 (* vs **), (*p*<0.001 (* vs ***),* p*<0.001 (** vs ***): Bonferroni test

*p*=0.140 (F vs C), *p*<0.001 (F vs N), *p*<0.001 (C vs N): Kruskal–Wallis test

Table 4 shows the relationship between age at onset and outcome of binocular response. No differences in positive binocular response between at near and at distance of the three groups based on ages at onset were found. However, both binocular positive response at near and at distance tended to be significantly better with older age at onset, from 83% and 87% to 99% (at near; p=0.009), and from 69% and 75% to 94% (at distance; p=0.001). From those results, the most important factor affecting better outcome of binocular response also appeared to be older age at onset.

**Table 4:** Comparisons of binocular response at final visit between groups based on age at onset

Age at onset	Binocular response		*P* values
	At near	At distance	(chi-squared test)
≤1 y (n=58)	48 (83%)	40 (69%)	0.083
>1 to ≤ 2 y (n=45)	39 (87%)	36 (75%)	0.396
> 2 y (n=68)	67 (99%)	64 (94%)	0.362
*P* value (Kruskal–Wallis test)	0.009	0.001	

*y* year(s)

Amblyopia was found in 45 patients (26%) during treatment, with no significant difference between bilateral (24 patients) and unilateral (21 patients). Frequencies of amblyopia in the 58 patients with age at onset ≤1 year old, in the 45 patients with age at onset >1 to ≤2 years old and in the 68 patients with age at onset >2 years old were 28% (16 patients), 33% (15 patients), and 21% (14 patients), respectively. Frequencies of amblyopia were 11% (4 patients) in the 37 patients of Group F, 30% (25 patients) in the 82 patients of Group C, and 31% (16 patients) in the 52 patients of Group N (Table 5). No significant relationship between presence of amblyopia and stereopsis outcome was found (p=0.054).

**Table 5:** Relationship between amblyopia and stereopsis outcome

Amblyopia	F: fine stereopsis	C: coarse stereopsis	N: nil stereopsis
	(n=37)	(n=82)	(n=52 )
(+): 45 patients	4 (11%)	25 (30%)	16 (31%)
(−): 126 patients	33 (89%)	57 (70%)	36 (69%)

*p*=0.054, Kruskal–Wallis test

According to a previous report by Fawcett and coworkers, duration of misalignment for 4 months was associated with outcome of stereopsis [11], and the relationship in outcomes of stereopsis for the 103 patients with age at onset ≤2 years between duration of misalignment ≤4 months (24 patients) and >4 months (79 patients) was investigated (Table 6). The rate of measurable stereopsis was significantly higher in patients with duration of misalignment ≤4 months (67%) than in patients with duration of misalignment >4 months (42%; p=0.032).

**Table 6:** Relationship between duration of misalignment and stereopsis outcome in patients with age at onset ≤ 2 years

103 patients with age at onset ≤ 2 years		
Duration of misalignment	Stereopsis (+)	Stereopsis (−)
≤ 4 months (n=24)	16 (67%)	8 (33%)
> 4 months (n=79)	33 (42%)	46 (58%)

*p*=0.032, Fisher’s exact test

## Discussion

Successful strabismus treatment is defined as alignment to within 8–10 PD of orthophoria in association with bi- or mono-fixation [4, 5]. With those ocular positions, favorable binocularity can be established. Although RAET is diagnosed if the deviation with full cycloplegic hyperopic correction was either eliminated or reduced to ≤10 PD at both near and distance fixation and favorable stereopsis might be expected after successful alignment, not all children with RAET achieve normal stereopsis after successful realignment with glasses, despite late onset after 5 years old [3, 6–19]. In our report, mean final stereopsis in all patients was 613" and fine stereopsis (≤60") and coarse stereopsis (>60" to ≤3000") were found in 37 patients (22%) and 82 patients (48%), respectively. However, another 52 children (30%) showed no stereopsis at the final visit. These results were disappointing, given that successful realignment with glasses was achieved in all patients with RAET in this report. A report by Fawcett and coworkers found that the distributions of fine stereopsis (≤60"), coarse stereopsis (>60" to ≤3000") and no stereopsis were 13%, 41% and 46% in 111 patients with RAET [8], not as satisfactory as in our report. Similarly, Berk and coworkers report that the distribution of fine stereopsis (≤100"), coarse stereopsis (>100" to ≤3000") and no stereopsis were 24%, 44% and 32% in 147 patients with RAET [13]. Lee and coworkers report that mean final stereopsis in 85 patients with RAET was 251.2", with fine stereopsis ≤100" identified in only 31 children (36.5%) [15]. From those reports, stereopsis outcomes in patients with RAET were not particularly good, even when successful realignment with glasses was obtained.

A critical question to resolve is what factors are associated with unsatisfactory stereopsis outcomes in patients with RAET showing successful realignment with glasses alone. This study compared clinical variables between Group F with fine stereopsis (≤60"), Group C with coarse stereopsis (>60" to ≤3000") and Group N with nil stereopsis. The refractive errors of both eyes, and angle of deviation without glasses both at near and distance at initial visit were not risk factors associated with stereopsis outcomes in patients with RAET. On the other hand, results of comparisons between the three groups showed that age at onset, age at initial visit, and age at first wearing glasses were critical factors associated with worse outcomes of stereopsis (Table 2). In particular, age at onset differed significantly between each of the three groups. Mean (± standard deviation) age at onset was significantly lower in Group N (nil stereopsis) (0.9 ± 0.6 years) than in Group F (3.1 ± 1.7 years, p<0.001) or Group C (2.2 ± 1.4 years, p<0.001). Further, age at onset was significantly older in Group F (fine stereopsis) than in Group C (coarse stereopsis) (p=0.010). In contrast, Lee and coworkers report different results, with no significant difference in age at presumed onset between children with good stereopsis (≤100": 3.4 ± 1.5 years) or poor stereopsis (>100": 2.9 ± 1.3 years; p=0.122) [15]. However, their poor stereopsis group was a mixture of what we defined as Groups C and N, and the differing definitions may well have contributed to these divergent results.

We investigated differences in stereopsis outcomes between three groups with ages at onset ≤1 year, >1 to ≤2 years, and >2 years (Table 3). The 68 patients with age at onset >2 years showed significantly better stereopsis at final visit (mean: 104") than the 45 patients with onset at >1 to ≤2 years (mean: 1403"; p<0.001) or the 58 cases with onset at ≤1 year (mean: 2581", p<0.001). Moreover, all children (100%) with age at onset >2 years showed measurable stereopsis, while incidences of measurable stereopsis in patients with age at onset ≤1 year or >1 year to ≤2 years were 40% and 62%, respectively. Coats and coworkers reported that incidences of measurable stereopsis in 17 patients with early onset before 1 year old and in 20 patients with onset after 2 years old were 89% (8 patients) and 80% (16 patients), showing no significant difference [18]. Berk and coworkers also report differing incidences of measurable stereopsis between 20 patients with early onset before 1 year old (60%, 12 patients) and 103 children with onset at 2–3 years old (70.8%, 73 patients) [13]. Although comparisons of the two measurable stereopsis outcomes with early onset by Berk and coworkers and our study using the chi-squared test showed no significant difference (p=0.195), our measurable stereopsis outcomes with onset >2 years were significantly better than the outcomes described by Coats and coworkers [18] or Berk and coworkers [13] (p=0.009 and p<0.001, respectively; Fisher’s exact test). Our results suggest that earlier age at onset with RAET is one of the most important factors associated with poor outcome of stereopsis.

Several studies demonstrated that the development of stereopsis starts abruptly at 3–4 months old, with maturation of stereopsis normally proceeding rapidly during the first year of life [25–29]. Thereafter, slower improvement in stereopsis continues beyond 18 months of age [27–29]. During this period, an abnormal binocular experience may lead to unfavorable prognosis for the restoration of normal stereopsis, even if ocular realignment is achieved with surgery or glasses [6]. Therefore, earlier surgery before 6–8 months old is recommended to develop favorable stereopsis in cases with infantile esotropia [6, 20]. Recent studies also report that the duration of misalignment plays a critical role in binocular sensory outcomes for children with RAET and suggest that the threshold duration is within 4 months [6, 9, 11]. In our report, no significant differences in duration of misalignment were evident between the three groups based on stereopsis outcomes (Table 2). Lee and coworkers also report no significant difference in duration of esodeviation between groups with good and poor stereopsis, similar to our results [15]. However, a significant relationship between duration of misalignment ≤4 months and stereopsis outcome was found for 103 patients with age at onset ≤2 years in our study (Table 6). Fawcett and coworkers suggest that periods of constant esotropia ≥4 months, due to failure of glasses treatment and/or noncompliance with wearing glasses, are associated with permanent stereo-deficits even in patients showing normal stereopsis at the onset of esotropia [12]. However, determining the exact duration of misalignment, success or failure of all-day glasses’ wearing and level of compliance with wearing glasses may be difficult. In this study, we determined the age at onset by the timing of initiation for the intermittent phase based on responses by parents or guardians and by photo-documentation. All patients with RAET have an intermittent phase of esotropia and show a transitional stage between intermittent and constant phases. This transitional stage varies between individuals, and such differences may produce better stereopsis outcomes in patients with duration of misalignment ≤4 months. At the same time, the influence of duration of misalignment on outcomes of stereopsis in patients with earlier onset should be investigated in future studies.

In this study, no major impact on outcomes of stereopsis in the presence of anisometropia, cycloplegic refractions at initial visit and at final visit, or amblyopia were found. However, significant differences in final near deviation with full corrective glasses were found between the three groups based on stereopsis outcome (Table 2).

Near and distant final deviations with full corrective glasses in three groups with age at onset ≤1 year, >1 to ≤2 years, and >2 years are shown in Table 7. Significant differences were evident in both near and distant final deviations between three groups with age at onset ≤1 year, >1 to ≤2 years, and >2 years (at near: 0.002, at distance: 0.043; one-way ANOVA). Multiple comparisons with Bonferroni testing revealed a significant difference only in near final deviation between groups with onset at >1 to ≤2 years and at >2 years (3.4 ± 3.9 PD vs 1.1 ± 3.9 PD, p=0.001). However, no difference in near final deviations was found between two groups with onset at ≤1 year old and at >2 years old. As mentioned before (Table 2), patients with onset at >2 years old showed significantly better stereopsis at final visit than the patients with onset at >1 to ≤2 years old or patients with onset at ≤1 year old. Those results of no difference in near final deviations between groups with onset at ≤1 year old and at >2 years old do not support the notion that better outcome of stereopsis was associated with smaller final near deviation with full corrective glasses. Lee and coworkers report that good stereopsis may only be achievable with misalignment ≤4 PD at distance and ≤5 PD at near fixation [15]. However, mean age at presumed onset in their report of 85 patients with RAET was 3.0 ± 1.4 years, similar to the 3.1 ± 1.7 years in 37 patients with fine stereopsis outcome in our study. Further study will be considered to clarify whether the same conclusions reached by Lee and coworkers can be obtained in patients with onset at <2 years old.

**Table 7:** Comparisons of final deviation with glasses between groups based on age at onset

Age at onset	Final deviation with glasses (PD)		*P* values
	At near	At distance	(t-test)
≤1 y (n=58)	2.6 ± 3.9^*^	2.2 ± 3.9^#^	0.602
>1 to ≤ 2 y (n=45)	3.4 ± 3.9^**^	3.4 ± 5.4^##^	1.000
> 2 y (n=68)	1.0 ± 2.1^***^	1.4 ± 3.0^###^	0.484
*P* value (one-way ANOVA)	0.002	0.043	

*PD* prism diopter, *y* year(s)

At near: *p*=0.647 (^*^ vs ^**^),* p*=0.044 (^*^ vs ^***^),* p*=0.001 (^**^ vs ^***^); Bonferroni test (significant:* p*<0.05/3)

At distance:* p*=0.412 (^#^ vs ^##^),* p*=0.866 (^#^ vs ^###^), *p*=0.037 (^##^ vs ^###^); Bonferroni test (significant: *p*<0.05/3)

Fawcett and Birch report that 75% of patients with RAET had anomalous binocular vision following successful eye alignment and suggested that constant eye misalignment lasting 4 months posed a significant risk for anomalous binocular vision on all measures studied, using measures of stereopsis, fusional vergence, sensory foveal fusion, and motion visual-evoked potential [11]. To improve outcomes of stereopsis in patients with earlier onset of RAET, future studies need to examine whether prompt correction of misalignment soon after initiation of deviation is necessary to develop better binocular vision and to obtain desirable final near deviation with full corrective glasses.

In conclusion, we have clarified that earlier age at onset represents one of the most important factors associated with poor outcome of stereopsis based on the relationship between outcomes of stereopsis and onset of misalignment in patients with RAET. In patients with infantile esotropia, although poor outcomes of stereopsis have remained a problem to be solved since the late 20^th^ century, surgery before 6–8 months old offers improved favorable stereopsis outcomes, sometimes achieving normal stereopsis [6, 20]. The shorter the interval between onset of infantile esotropia and timing of surgical realignment, the better the outcomes of stereopsis. For the prognosis of stereopsis in RAET, the same problem may be associated with improvements in stereopsis outcomes. A prompt initial visit soon after esotropic ocular deviation may be the key to minimizing the duration of misalignment among infants with RAET. We advocate prompt consultation regarding early-onset strabismus for parents or guardians to receive realignment with surgery or glasses.
